# Supplementary material for: A Cross-Sectional Study on Canine and Feline Anal Sac Disease
Source: Animals (Basel). 2021 Dec 31;12(1):95. doi: 10.3390/ani12010095 (PMC8749694; doi:10.3390/ani12010095)
Supplement: Supplementary file 1 [file animals-12-00095-s001.zip › animals-1526536-supplementary.pdf]

S1 Questionnaires

Dog questionnaire

Survey concerning canine anal sac disease

This survey is made as part of an observational study on anal sac disease in the dog at the University of Utrecht, supervised by Dr. R.J. Corbee. The data will be used to determine the prevalence and predisposing factors and to clarify how the diagnosis is made and which therapy is used in several types of anal sac disease of the dog. There is also a similar survey concerning anal sac disease in the cat.

By completing this survey, you are contributing to a better understanding of the prevalence, predisposing factors, diagnosis and therapy of this disease in dogs.

The survey consists of multiple-choice questions and descriptive questions. Each page contains a maximum of six questions and the survey has a total of ten pages. During the survey it is possible to return to the previous page to review your answer.

For the quality of the survey, it is important that you make sure to answer all of the questions completely and that you fill in the survey only once. Your personal data will be handled with great care and will be deleted after processing the data.

The survey will take approximately ten to fifteen minutes.

If you have any questions or if anything is unclear, you can contact Hilde Woldring.

Thank you in advance for your participation!

Q2 Prevalence of anal sac disease in the dog

In this section, questions will be asked about the prevalence of anal sac disease in the dog. If you do not know the exact numbers, an estimation can be made.

Q3

How many dogs did you treat in the last year (between September 2019 and September 2020)?

____

Q4

How many dogs did you diagnose with anal sac disease (impaction, inflammation or abscessation) in the last year (between September 2019 and September 2020)?

____

Q6

How many dogs did you diagnose with an anal sac impaction in the last year (between September 2019 and September 2020)?

____

Q48

Do dogs with anal sac impaction get presented to the veterinary assistant?

o Yes: how many dogs with anal sac impaction were presented to the veterinary assistant in the last year (between September 2019 and September 2020)? (1) ____

o No (2)

Q7

How many dogs did you diagnose with an anal sac inflammation in the last year (between September 2019 and September 2020)?

____

Q9 How many dogs did you diagnose with anal sac abscessation in the last year (between September 2019 and September 2020)?

____

Q10 Predisposing factors for anal sac disease in the dog

In this section, questions will be asked about predisposing factors for anal sac disease in the dog.

Q11 Anal sac disease (such as impaction, inflammation or abscessation) is more often seen in:

o Male dogs (1)

o Female dogs (2)

o No difference (3)

Q12

Anal sac disease (such as impaction, inflammation or abscessation) is more often seen in:

o Uncastrated male dogs (1)

o Castrated male dogs (2)

o No difference (3)

Q13 Anal sac disease (such as impaction, inflammation or abscessation) is more often seen in:

o Uncastrated female dogs (1)

o Castrated female dogs (2)

o No difference (3)

Q14 Anal sac disease (such as impaction, inflammation or abscessation) is more often seen in:

o Young dogs ((1)

o Mature dogs/old dogs (>1 year) (2)

o No difference (3)

o Other, please specify... (4) ____

Q15 Anal sac disease (such as impaction, inflammation or abscessation) is more often seen in:

o Obese dogs (body condition score of: 7 or higher on a 9 point scale / 4 or higher on a 5 point scale) (1)

o Non-obese dogs (body condition score of: 6 or lower on a 9 points scale / 3 or lower on a 5 points scale) (2)

o No difference (3)

Q39 Anal sac disease (such as impaction, inflammation or abscessation) is more often seen in:

o Small dogs (< 10 kg) (1)

o Medium sized dogs (between 10 and 30 kg) (2)

o Large dogs (> 30 kg) (3)

o No difference (4)

Q16

Anal sac disease (such as impaction, inflammation or abscessation) is more often seen in certain breeds, such as:

- ▢ Chihuahua (1)
- ▢ Miniature poodle (2)
- ▢ Lhasa Apso (3)
- ▢ Jack Russell Terrier (4)
- ▢ French bulldog (5)
- ▢ Beagle (6)
- ▢ Staffordshire Bull Terrier (7)
- ▢ Labrador retriever (8)
- ▢ German shepherd (9)
- ▢ Another breed or other breeds, namely... (10)

____

- ▢ No difference (11)

Q17 Anal sac disease (such as impaction, inflammation or abscessation) is more often seen in dogs with a certain type of coat, such as:

- o Short coat (1)
- o Long coat (2)
- o No difference (3)
- o Other, please specify... (4) ____

Q18

Anal sac disease (such as impaction, inflammation or abscessation) is more often seen in dogs on a certain type of diet, such as:

- o Dry food (kibble), namely... (1) ____
- o Wetfood, namely... (2) ____
- o A combination of dry- and wetfood (3)
- o BARF: Bone And Raw Food, namely... (4)

____

- o Home made diet (5)
- o No difference (6)
- o Other, please specify... (7) ____

Q48

Anal sac disease (such as impaction, inflammation or abscessation) is more often seen in dogs in a certain season:

o Yes, namely... (1) ____

o No (2)

Q19

Anal sac disease (such as impaction, inflammation or abscessation) is more often seen in dogs with a skin condition, such as:

▢ The presence of ectoparasites (flea, lice, mites, ticks) (1)

▢ Flea hypersensitivity or flea allergy dermatitis (2)

▢ Cutaneous adverse food reaction or food allergy (3)

▢ Allergic contact dermatitis (4)

▢ Hypersensitivity induced by insects bites, namely... (5)
____

▢ Atopic dermatitis (6)

▢ Malassezia dermatitis (7)

▢ Primary superficial pyoderma (8)

▢ Other, please specify... (9) ____

▢ No difference (10)

Q20

Anal sac disease (such as impaction, inflammation or abscessation) is more often seen in dogs with a gastrointestinal condition, such as:

▢ Viral or bacterial enteritis (1)

▢ Worm infection (2)

▢ Gastritis (3)

▢ Cutaneous adverse food reaction (4)

▢ Neoplasia (5)

▢ Other, please specify... (6) ____

▢ No difference (7)

Q56 Anal sac disease (such as impaction, inflammation or abscessation) is more often seen in dogs with diarrhea:

o Yes, namely in small bowel diarrhea (1)

o Yes, namely in large bowel diarrhea (2)

o Yes, but no difference in small- or large bowel diarrhea (3)

o No (4)

Q21 Diagnosis

In this section, questions will be asked about the criteria on which the diagnosis of anal sac disease in the dog is based.

Q22

The criterion or the combination of criteria on which your diagnosis of anal sac disease (such as impaction, inflammation or abscessation) in the dog is based, is/are:

Multiple answers possible.

▢ The presence of one or more of the following clinical symptoms: licking or biting the anal area frequently, scooting, tail chasing, tenesmus, rubbing the anal area against objects, discomfort when sitting down and/or perianal discharge (1)

▢ The size of the anal sac (2)

▢ The consistency of the anal sac (3)

▢ The shape of the anal sac (4)

▢ The temperature of the anal sac (5)

▢ The color of the anal area (6)

▢ The presence of pain in palpation of the anal sac (7)

▢ The ease by which the anal sac can be emptied (8)

▢ The amount of the anal sac contents (9)

▢ The consistency of the anal sac contents (10)

▢ The nature of the anal sac contents (11)

▢ The color of the anal sac contents (12)

▢ The smell of the anal sac contents (13)

▢ The body temperature (14)

▢ The reaction on inserting the thermometer (15)

▢ Microscopic examination of the anal sac contents (including the presence of bacteria, the amount of bacteria and the presence of polymorphonuclear leukocytes and/or erythrocytes) (16)

▢ Other, please specify... (17) ____

Q23

The distinction between anal sac impaction and anal sac inflammation in the dog, is based on:

____

Q40

The distinction between anal sac inflammation and anal sac abscessation in the dog, is based on:

____

Q24

The distinction between anal sac impaction and anal sac abscessation in the dog, is based on:

____

Q26 Treatment

In this section questions will be asked about the treatment of anal sac disease in the dog.

Q27

What treatment do you give dogs with anal sac impaction?

Multiple answers possible.

▢ Nothing/no treatment (1)

▢ Emptying the anal sacs by manual evacuation (2)

▢ Flushing the anal sacs, using… (3) ____

▢ Putting a local antibiotic (ointment) in the anal sacs, namely... (4)

____

▢ Prescribing a systemic antibiotic, namely… (5)

____

▢ Treating a potential, underlying cause (6)

▢ This depends on the severity of the condition, namely... (7)

____

▢ Other, please specify... (8) ____

Q58

What treatment do you give dogs with anal sac inflammation?

Multiple answers possible.

▢ Nothing/no treatment (1)

▢ Emptying the anal sacs by manual evacuation (2)

▢ Flushing the anal sacs, using… (3) ____

▢ Putting a local antibiotic (ointment) in the anal sacs, namely... (4)
____

▢ Prescribing a systemic antibiotic, namely… (5)
____

▢ Treating a potential, underlying cause (6)

▢ This depends on the severity of the condition, namely... (7)
____

▢ Other, please specify... (8) ____

Q59

What treatment do you give dogs with anal sac abscessation?

Multiple answers possible.

▢ Nothing/no treatment (1)

▢ Emptying the anal sacs by manual evacuation (2)

▢ Flushing the anal sacs, using… (3) ____

▢ Putting a local antibiotic (ointment) in the anal sacs, namely... (4)
____

▢ Prescribing a systemic antibiotic, namely… (5)
____

▢ Treating a potential, underlying cause (6)

▢ This depends on the severity of the condition, namely... (7)
____

▢ Other, please specify... (8) ____

Q30

Do you apply the same treatment in a cat with anal sac impaction?

o Yes (1)

o No, please specify... (2) ____

Q31 Do you apply the same treatment in a cat with anal sac inflammation?

o Yes (1)

o No, please specify... (2) ____

Q33 Do you apply the same treatment in a cat with anal sac abscessation?

o Yes (1)

o No, please specify... (2) ____

Q43 When do you decide to surgically remove the anal sacs of a dog?

____

____

____

____

____

Q44 What technique do you use for the surgical removal of the anal sacs of a dog?

▢ Open technique (1)

▢ Closed technique (2)

▢ Other, please specify... (3) ____

Q45 Can you please give a short description on how you perform this technique?

____

____

____

____

____

Q34

Effectiveness of the treatment

In this section, questions will be asked about the effectiveness of the treatment of anal sac disease in the dog in the last year (between September 2019 and September 2020).

Q35

How many dogs with anal sac disease (such as impaction, inflammation or abscessation) have relapsed after treatment in the last year (between September 2019 and September 2020)?

____

____

____

____

____

Q42

On average, in what time frame does a relapse occur after the treatment of an anal sac disease (such as impaction, inflammation or abscessation) in dogs?

____

Q52

On average, in what time frame does a relapse occur after the treatment of an anal sac impaction in dogs?

____

Q53

On average, in what time frame does a relapse occur after the treatment of an anal sac inflammation in dogs?

____

Q54

On average, in what time frame does a relapse occur after the treatment of anal sac abscessation in dogs?

____

Q36

How many dogs with an anal sac impaction have relapsed after treatment in the last year (between September 2019 and September 2020)?

____

____

____

____

____

Q37 How many dogs with an anal sac inflammation have relapsed after treatment in the last year (between September 2019 and September 2020)?

____

____

____

____

____

Q38 How many dogs with anal sac abscessation have relapsed after treatment in the last year (between September 2019 and September 2020)?

____

____

____

____

____

Q41 In conclusion In this final section you can specify whether you would like to receive the end results of this research and you can share any comments or recommendations for this survey. Furthermore, we ask you when you graduated, in which country you work and whether you would like to participate in the survey for the cat.

Q47 Do you have any comments, recommendations or tips to improve this survey?

____

____

____

____

____

Q42 Would you like to receive the final report, including the results of this study?

o Yes, this is my e-mail address: (1) ____

o No thanks (2)

Q55 In which year did you graduate?

____

Q57 In which country do you work?

____

____

____

____

____

Q41 In addition to this study on anal sac disease in the dog, there is also a similar study on anal sac disease in the cat. Would you also like to participate in this survey? You would help us a lot!

You can copy this link: https://survey.uu.nl/jfe/form/SV_bfHuDFojwVZZIgt

Cat questionnaire

Survey concerning feline anal sac disease in English

This survey is made as part of an observational study on anal sac disease in the cat at the University of Utrecht, supervised by Dr. R.J. Corbee. The data will be used to determine the prevalence and predisposing factors and to clarify how the diagnosis is made and which therapy is used in several types of anal sac disease of the cat. There is also a similar survey concerning anal sac disease in the dog. With completing this survey, you are contributing to a better understanding of the prevalence, predisposing factors, diagnosis and therapy of this disease in cats.

The survey consists of multiple choice questions and descriptive questions. Each page contains a maximum of six questions and the survey has a total of ten pages. During the survey it is possible to return to the previous page to review your answer. For the quality of the survey, it is important that you make sure to answer all of the questions completely and that you fill in the survey only once. Your personal data will be handled with great care and will be deleted after processing the data. The survey will take approximately ten to fifteen minutes.

If you have any questions or if anything is unclear, please contact Lianne van den Eijnde.

Thank you in advance for your participation!

Section 1. Prevalence of anal sac disease in the cat

In this section, questions will be asked about the prevalence of anal sac disease in the cat. If you do not know the exact numbers, an estimation can be made.

1. How many cats did you treat in the last 4 years (between September 2017 and September 2020)?

2. Do you work in a cat clinic / a cat friendly clinic?

Yes

No

3. How many cats did you diagnose with anal sac disease (impaction, inflammation or an abscess) in the last 4 years (between September 2017 and September 2020)?

4. How many cats did you diagnose with an anal sac impaction in the last 4 years (between September 2017 and September 2020)?

5. How many cats did you diagnose with an anal sac inflammation in the last 4 years (between September 2017 and September 2020)?

6. How many cats did you diagnose with an anal sac abscess in the last 4 years (between September 2017 and September 2020)?

Predisposing factors for anal sac disease in the cat

In this section, questions will be asked about predisposing factors for anal sac disease in the cat. In case the prevalence of cats with anal sac disease in the previous section is very low, you can answer the following questions based on all of the cats with anal sac disease that you have encountered during your entire career as a veterinarian.

7. Anal sac disease (such as impaction, inflammation or an abscess) is more often seen in:

Male cats

Female cats

No difference

8. Anal sac disease (such as impaction, inflammation or an abscess) is more often seen in:

Uncastrated male cats

Castrated male cats

No difference

9. Anal sac disease (such as impaction, inflammation or an abscess) is more often seen in:

Uncastrated female cats

Castrated female cats

No difference

10. Anal sac disease (such as impaction, inflammation or an abscess) is more often seen in:

Young cats (<3 year)

Mature cats (between 3 and 10 years)

Old cats (>10 years)

No difference

Other, please specify: …

11. Anal sac disease (such as impaction, inflammation or an abscess) is more often seen in:

Obese cats (body condition score of: 7 or higher on a 9 point scale / 4 or higher on a 5 point scale)

Non-obese cats (body condition score of: 6 or lower on a 9 point scale / 3 or lower on a 5 point scale)

No difference

12. Anal sac disease (such as impaction, inflammation or an abscess) is more often seen in certain breeds, such as:

British shorthair

European shorthair

Maine Coon

Norwegian forest cat

Persian cat

Siamese

Sphynx

Other, please specify: ...

No difference

13. Anal sac disease (such as impaction, inflammation or an abscess) is more often seen in cats with a certain type of coat, such as:

Short coat

Long coat

Hairless

Other, please specify: ...

No difference

14. Anal sac disease (such as impaction, inflammation or an abscess) is more often seen in cats on a certain type of diet, such as:

Dry food (kibble), namely...

Wet food, namely...

A combination of dry- and wet food, namely...

BARF: bone and raw food, namely...

Home made diet

Other, please specify: …

No difference

15. Anal sac disease (such as impaction, inflammation or an abscess) is more often seen in cats in a certain season:

Yes, namely...

No difference

16. Anal sac disease (such as impaction, inflammation or an abscess) is more often seen in cats with a skin condition, such as:

The presence of ectoparasites (flea, lice, mites, ticks)

Flea hypersensitivity or flea allergy dermatitis

Cutaneous adverse food reaction or food allergy

Allergic contact dermatitis

Hypersensitivity induced by insect bites, namely…

Atopic dermatitis

Malassezia dermatitis

Primary superficial pyoderma

Other, please specify: …

No difference

17. Anal sac disease (such as impaction, inflammation or an abscess) is more often seen in cats with a gastrointestinal condition, such as:

Viral or bacterial enteritis

Worm infection

Gastritis

Cutaneous adverse food reaction

Neoplasia

Other, please specify: …

No difference

18. Anal sac disease (such as impaction, inflammation or an abscess) is more often seen in cats with diarrhea:

Yes, namely in small intestinal diarrhea

Yes, namely in large intestinal diarrhea

Yes, but no difference in small- or large intestinal diarrhea

No

Diagnosis

In this section, questions will be asked about the criteria on which the diagnosis of anal sac disease in the cat is based.

19. The symptoms(s) that can be observed in a cat with anal sac disease (such as impaction, inflammation or an abscess), is/are:

Multiple answers possible.

Frequently licking or biting the anal region

Frequently licking or biting the tail region

Tail chasing

Scooting

Rubbing the anal area against objects

Discomfort when sitting down

Perianal discharge

Tenesmus

Other, please specify: ...

20, The criterion or the combination of criteria on which your diagnosis of anal sac disease (such as impaction, inflammation or an abscess) in the cat is based, is/are:

Multiple answers possible.

The presence of one or more of the following clinical symptoms: licking or biting the anal area frequently, scooting, tail chasing, tenesmus, rubbing the anal area against objects, discomfort when sitting down and/or perianal discharge

The size of the anal sac

The consistency of the anal sac

The shape of the anal sac

The temperature of the anal sac

The color of the anal area

The presence of pain in palpation of the anal sac

The ease by which the anal sac can be emptied

The amount of the anal sac contents

The consistency of the anal sac contents

The nature of the anal sac contents

The color of the anal sac contents

The smell of the anal sac contents

The body temperature

The reaction on inserting the thermometer

Microscopic examination of the anal sac contents (including the presence of bacteria, the amount of bacteria and the presence of polymorphonuclear leukocytes and/or erytrocytes)

Other, please specify...

21. The distinction between anal sac impaction and anal sac inflammation in the cat, is based on:

22. The distinction between anal sac inflammation and an anal sac abscess in the cat, is based on:

23. The distinction between anal sac impaction and an anal sac abscess in the cat, is based on:

Treatment

In this section questions will be asked about the treatment of anal sac disease in the cat.

24. What treatment do you give cats with anal sac impaction?

Multiple answers possible.

Nothing/no treatment

Emptying the anal sacs by manual evacuation

Flushing the anal sacs, using…

Putting a local antibiotic (ointment) in the anal sacs, namely...

Prescribing a systemic antibiotic, namely…

Surgical removal of the anal sacs

Treating a potential, underlying cause

This depends on the severeness of the condition, namely...

Other, please specify: ...

25. What treatment do you give cats with anal sac inflammation?

Multiple answers possible.

Nothing/no treatment

Emptying the anal sacs by manual evacuation

Flushing the anal sacs, using…

Putting a local antibiotic (ointment) in the anal sacs, namely...

Prescribing a systemic antibiotic, namely…

Surgical removal of the anal sacs

Treating a potential, underlying cause

This depends on the severeness of the condition, namely...

Other, please specify: ...

26. What treatment do you give cats with an anal sac abscess?

Multiple answers possible.

Nothing/no treatment

Emptying the anal sacs by manual evacuation

Flushing the anal sacs, using…

Putting a local antibiotic (ointment) in the anal sacs, namely...

Prescribing a systemic antibiotic, namely…

Surgical removal of the anal sacs

Treating a potential, underlying cause

This depends on the severeness of the condition, namely…

Other, please specify: ...

27. Do you apply the same treatment in a dog with anal sac impaction?

Yes

No, please specify: ...

28. Do you apply the same treatment in a dog with anal sac inflammation?

Yes

No, please specify: ...

29. Do you apply the same treatment in a dog with an anal sac abscess?

Yes

No, please specify: ...

30. When do you decide to surgically remove the anal sacs of a cat?

31. What method do you use for the surgical removal of the anal sacs of a cat?

Open method

Closed method

Other, please specify: ...

32. Can you please give a short description on how you perform this method?

Section 5. Effectiveness of the treatment

In this section, questions will be asked about the effectiveness of the treatment of anal sac disease in the cat.

33. How many cats with anal sac disease (such as impaction, inflammation of abscess) have relapsed after treatment in the last 4 years (between September 2017 and September 2020)?

34. How many cats with anal sac impaction have relapsed after treatment in the last 4 years (between September 2017 and September 2020)?

35. How many cats with anal sac inflammation have relapsed after treatment in the last 4 years (between September 2017 and September 2020)?

36. How many cats with an anal sac abscess have relapsed after treatment in the last 4 years (between September 2017 and September 2020)?

37. On average, in what time frame does a relapse occur after the treatment of an anal sac disease (such as impaction, inflammation or abscess) in cats?

38. On average, in what time frame does a relapse occur after the treatment of an anal sac impaction in cats?

39. On average, in what time frame does a relapse occur after the treatment of an anal sac inflammation in cats?

40. On average, in what time frame does a relapse occur after the treatment of an anal sac abscess in cats?

Section 6. In conclusion

In this final section you can specify whether you would like to receive the end results of this research and you can share any comments or recommendations for this survey. Furthermore, we ask you when you graduated, in which country you work and whether you would like to participate in the survey for the dog.

41. Do you have any comments, recommendations or tips to improve this survey?

42. Would you like to receive the final report, including the results of this study?

Yes, this is my e-mail address: …

No thanks

43. In which year did you graduate?

44. In which country do you work?

45. In addition to this study on anal sac disease in the cat, there is also a similar study on anal sac disease in the dog. Would you also like to participate in this survey? You would help us out a lot!

You can copy this link: https://survey.uu.nl/jfe/form/SV_7PYNb4lGxRlABmZ

S2 Additional results

Distinguish between impaction, inflammation, abcessation

Criteria used for the distinction between anal sac impaction and anal sac inflammation in dogs by 54 participants:

anal sac content (not further specified) (n=18), presence of discomfort or pain (n=18), consistency of the anal sac content (n=14), color of the anal sac content (n=11), presence of signs of inflammation of the anal area (n=8), type of anal sac content (n=7), odor of the anal sac content (n=7), consistency of the anal sac (n=6), ease of emptying the anal sac (n=5), presence of clinical signs (n=4), volume within the anal sac (n=4), thickness of the anal sac wall (n=3), size of the anal sac (n=2), amount of anal sac content (n=2), body temperature (n=2), findings on clinical examination (n=1), shape of the anal sac (n=1), cytology of the anal sac content (n=1).

Criteria used for the distinction between anal sac impaction and anal sac inflammation in cats by 49 participants:

pain during palpation of the anal sac (n=18), nature of anal sac contents (n=17), consistency of anal sac contents (n=14), anal sac content (not further specified) (n=11), color of the anal sac content (n=10), perianal swelling (n=7), ease of emptying the anal sac (n=5), volume within the anal sac (n=4), color of the anal area (n=4), thickness of the anal sac wall (n=3), consistency of the anal sac (n=2), findings on clinical examination (n=2), body temperature (n=1), cytology of the anal sac content (n=1).

Criteria used for the distinction between anal sac inflammation and anal sac abscessation in dogs by 54 participants:

fistula formation (n=25), type of anal sac content (n=18), presence of discomfort or pain (n=11), size of the anal sac (n=10), ease of emptying the anal sac (n=10), anal sac content (not further specified) (n=10), presence of signs of inflammation of the anal area (n=9), shape of the anal sac (n=7), consistency of the anal sac (n=6), volume within the anal sac (n=5), presence of clinical signs (n=3), findings on clinical examination (n=2), body temperature (n=2), thickness of the anal sac wall (n=1), consistency of the anal sac content (n=1).

Criteria used for the distinction between anal sac inflammation and anal sac abscessation in cats by 48 participants:

type of anal sac content (n=17), fistula formation (n=15), size of the anal sac (n=11), presence of discomfort or pain (n=9), presence of a wound (n=9), perianal swelling (n=7), anal sac content (not further specified) (n=3), ease of emptying the anal sac (n=3), thickness of the anal sac wall (n=2), consistency of the anal sac content (n=2), color of anal area (n=2), findings on clinical examination (n=1), body temperature (n=1), cytology of the anal sac content (n=1), color of the anal sac content (n=1), smell of the anal sac content (n=1).

Criteria used for the distinction between anal sac impaction and abscessation in dogs by 54 participants:

anal sac content (not further specified) (n=16), type of anal sac content (n=15), presence of discomfort or pain (n=15), presence of clinical signs (n=11), fistula formation (n=11), ease of emptying the anal sac (n=9), size of the anal sac (n=7), presence of signs of inflammation of the anal area (n=7), color of the anal sac content (n=6), consistency of the anal sac (n=5), thickness of the anal sac wall (n=5), consistency of the anal sac content (n=4), volume within the anal sac (n=3), body

temperature (n=3), amount of anal sac content (n=2), shape of the anal sac (n=2), odor of the anal sac content (n=2), cytology of the anal sac content (n=1).

Criteria used for the distinction between anal sac impaction and abscessation in cats by 48 participants:

type of anal sac content (n=16), presence of discomfort or pain (n=13), anal sac content (not further specified) (n=11), size of the anal sac (n=8), consistency of the anal sac content (n=6), perianal swelling (n=5), fistula formation (n=5), presence of a wound (n=5), color of the anal sac content (n=4), thickness of the anal sac wall (n=3), ease of emptying the anal sac (n=3), color of anal area (n=3), odor of the anal sac content (n=3), body temperature (n=3), temperature of the anal sac (n=1), cytology of the anal sac content (n=1).

Type of flush used

For anal sac impaction: isotonic saline solution (n=4 in dogs, n=7 in cats), povidone-iodine solution (n=1 in dogs), and chlorhexidine solution (n=1 in dogs, n=1 in cats).

For anal sac inflammation: isotonic saline solution (n=10 in dogs, n=11 in cats), povidone-iodine solution (n=6 in dogs, n=3 in cats), chlorhexidine solution (n=1 in dogs, n=1 in cats), and water (n=1 in cats).

For anal sac abscessation: isotonic saline solution (n=10 in dogs, n=12 in cats), povidone-iodine solution (n=10 in dogs, n=4 in cats), chlorhexidine solution (n=4 in dogs, n=3 in cats), and water (n=1 in cats).

Type of antibiotic ointment used

For anal sac impaction: chloramphenicol (n=1 in dogs), and polymyxin B sulphate (n=1 in dogs)

For anal sac inflammation: chloramphenicol (n=12 in dogs, n=6 in cats), polymyxin B sulphate (n=1 in dogs, n=2 in cats), silver sulfadiazine (n=2 in dogs, n=1 in cats), amoxicillin (or amoxicillin clavulanic acid) (n=1 in dogs, n=1 in cats) and duplocillin (n=1 in cats).

For anal sac abcessation: chloramphenicol (n=11 in dogs, n=6 in cats), amoxicillin (or amoxicillin clavulanic acid) (n=2 in dogs, n=1 in cats), a combination of benzylpenicillin, streptomycin and nafcillin (n=1 in dogs), cloxacillin (n=1 in dogs), gentamycin (n=1 in dogs), and duplocillin (n=1 in cats).

Type of systemic antibiotics prescribed

For anal sac impaction: quinolones (n=1 in dogs)

For anal sac inflammation: spiramycin and metronidazole (n=14 in dogs, n=14 in cats), metronidazole (n=3 in dogs, n=5 in cats), amoxicillin (or amoxicillin clavulanic acid) (n=2 in dogs, n=1 in cats), clindamycin (n=1 in dogs), and quinolones (n=1 in dogs).

For anal sac abcessation: piramycin and metronidazole (n=26 in dogs, n=17 in cats), metronidazole (n=8 in dogs, n=8 in cats), amoxicillin and clavulanic acid (n=17 in dogs, n=6 in cats), clindamycin (n=2 in dogs, n=1 in cats), and trimethoprim and sulfamethoxazole (TMP/S) (n=2 in dogs).
